# Pylephlebitis With Hepatic Abscess Complicating a Case of Acute Pancreatitis in a Young Male: Startling Complication of Intra-Abdominal Sepsis

**DOI:** 10.7759/cureus.21288

**Published:** 2022-01-16

**Authors:** Dhruv Talwar, Sourya Acharya, Samarth Shukla, Sunil Kumar, Akhilesh Annadatha

**Affiliations:** 1 Department of Medicine, Jawaharlal Nehru Medical College, Datta Meghe Institute of Medical Sciences (Deemed to be University), Wardha, IND; 2 Department of Pathology, Jawaharlal Nehru Medical College, Datta Meghe Institute of Medical Sciences (Deemed to be University), Wardha, IND

**Keywords:** pylephlebitis, thrombosis, portal vein, hepatic abscess, acute pancreatitis

## Abstract

The thrombophlebitis of the portal vein and or its hepatic branches accompanied with a positive blood culture is known as pylephlebitis, with the most common cause as sepsis. Liver abscess formation resulting from pylephlebitis is a rare but potentially lethal complication. We report a case of a 30-year-old male who presented with acute pain in the abdomen along with nausea and vomiting for two days with a history of alcohol dependence. Upon investigation, it was revealed to be a case of acute pancreatitis with hepatic abscess with superior mesenteric and portal vein thrombosis. The patient was managed conservatively with antibiotics and anticoagulants successfully. This case highlights the importance of keeping pylephlebitis with a hepatic abscess as a crucial potential complication of acute pancreatitis as its management promptly prevents mortality.

## Introduction

Waller described pylephlebitis as the source of liver abscess in the year 1846. Pylephlebitis (suppurative, infected portal vein thrombosis (PVT)) is rare to encounter but a dangerous complication arising out of intra-abdominal or pelvic infection and is often associated with significant morbidity as well as mortality [1]. Noncirrhotic and nonmalignant thrombosis of the portal vein has inconclusive etiology in about 25% of the cases. Pylephlebitis is the infective thrombosis of the portal vein, which was considered universally fatal in the pre-antibiotic era. With the emergence of modern imaging modalities and antibiotics, the outcome of pylephlebitis has improved significantly. Pylephlebitis can complicate any intra-abdominal or pelvic infection that affects the region drained by the portal vein. The most common intraabdominal infections to be associated with pylephlebitis are appendicitis and diverticulitis [2]. Most commonly pylephlebitis is associated with polymicrobial infections followed by monomicrobial infections with Streptococcus viridans, Bacteroides fragilis, as well as Escherichia coli [1].

Amongst the reported cases of pylephlebitis with liver abscess, pancreatitis is a rare cause, with only 5% of the reported cases having pancreatitis as the etiology [3]. Initially, pylephlebitis starts off as thrombophlebitis of veins draining the mesentery, which then spreads to the portal system and then the liver. Thrombosis of the mesenteric vein might result in ischemia and infarction of the mesentery as well as necrosis of the bowel. Risk factors that might increase the chances of pylephlebitis are hypercoagulable state, deficiency of clotting factors, smoking, history of abdominal surgical procedure, steroid use, coronary artery disease, prior history of cerebrovascular episode, deep vein thrombosis and cirrhosis.

The major challenge in treating pylephlebitis remains non-specific presentation of the condition. Patients usually present with pain in the abdomen, malaise, fever and nausea [4].

The imaging modality of choice in pylephlebitis is computed tomography which shows filling defects in the portal vein. While suspecting the diagnosis of pylephlebitis empirical antibiotic coverage should be initiated including metronidazole, gentamicin, piperacillin, ceftizoxime, imipenem, and ampicillin [5], which can be modified later on the basis of culture reports and treatment can be extended up to four to six weeks.

## Case presentation

**Figure 1:**
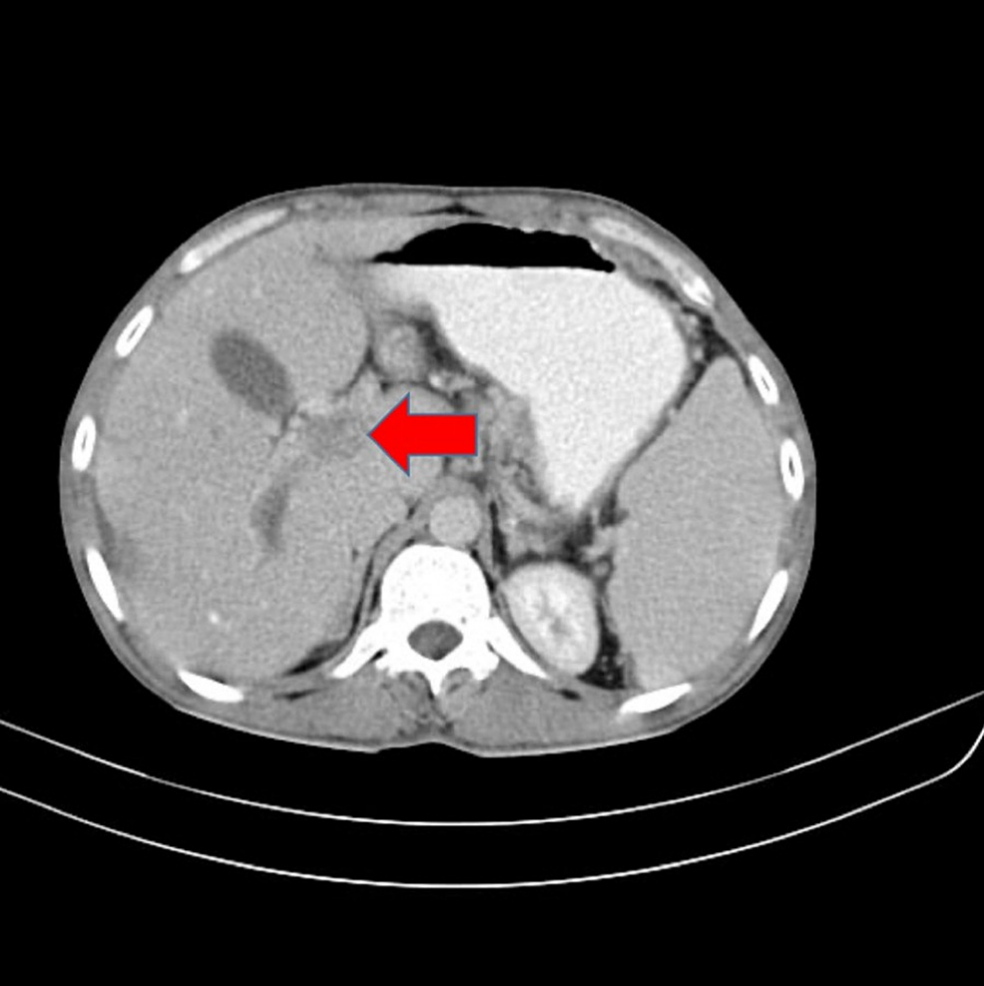
Contrast-enhanced computed tomography of the abdomen showing thrombosis of the main portal vein

**Figure 2:**
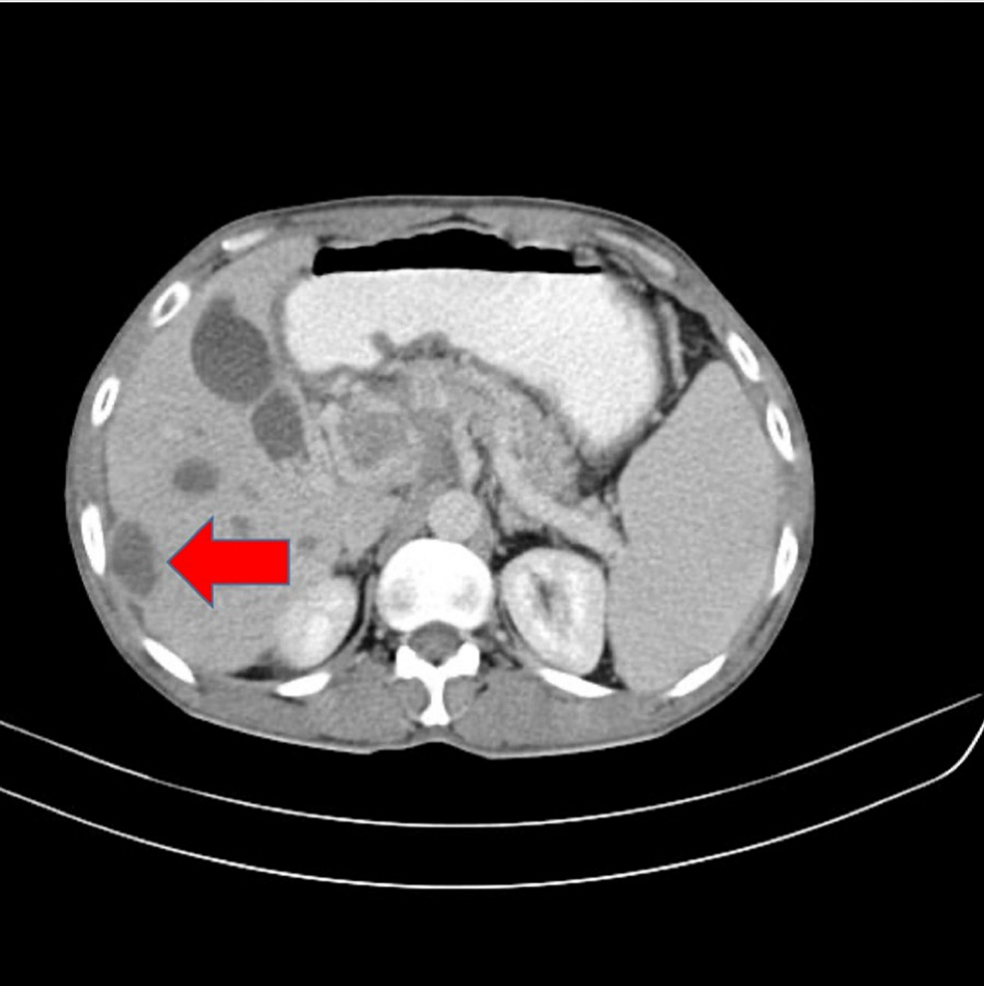
Contrast-enhanced computed tomography of the abdomen showing right subcapsular hepatic abscess

A 30-year-old male presented to the emergency department with a history of pain in the abdomen, fever, nausea and vomiting for two days. The patient had a history of alcohol abuse with the last intake four days back. His CAGE score was calculated to be 2. There was no history of hypertension, diabetes mellitus, coronary artery disease, deep vein thrombosis, cerebrovascular episode or any other chronic medical conditions in the past. On general examination, the patient was stable with a pulse of 82 beats per minute, blood pressure of 120/70 mm of hg in right arm supine position and SpO2 of 97% on room air. On systemic examination, the abdomen was soft with tenderness present in the epigastrium and the right hypochondrium and spleen was palpable. Heart sounds were normal, the chest was bilaterally clear and the patient was conscious and oriented. The patient was admitted for further evaluation and blood investigations were suggestive of acute pancreatitis with sepsis (Table 1). A contrast-enhanced computed tomography (CECT) of the abdomen was carried out, which was suggestive of acute pancreatitis with the portal and superior mesenteric vein thrombosis with hepatic abscess (Figure 1 and Figure 2). Ultrasonography was done by the interventional radiology team to assess the abscess for drainage and the abscess was found to be less than 5 cm in size and was advised to be managed conservatively. The patient was kept nil by mouth and started on empirical antibiotics (injectable metronidazole 100ml intravenous thrice a day and injectable piperacillin and tazobactam 4.45gm intravenous thrice a day) with anti-coagulants (tablet nicoumalone 2mg once a day). During the course of the hospital stay, the patient improved clinically and amylase lipase decreased. The patient's blood culture was positive for Klebsiella pneumoniae. He was allowed an oral diet on day 5 of admission and was finally discharged in stable condition seven days after admission. The patient was continued on tablet ciprofloxacin 500mg once a day for six weeks after discharge. Anticoagulation was continued for a duration of six months post discharge.

**Table 1:** Laboratory investigations of the case

Lab Investigation	Measures Value
Hemoglobin	9.8 gm/dl
Platelet count	1.17 lakh/dl
White blood cell count	14500/dl
Mean Corpuscular Volume	80 fl
Creatinine	0.8 mg/dl
Urea	27 mg/dl
Sodium	135 mmol/l
Potassium	4.4 mmol/l
Total Protein	6.4 gm/dl
Albumin	2.7 gm/dl
Globulin	3.7 gm/dl
Aspartate Aminotransferase	88 units/l
Alanine Aminotransferase	89 units/l
Alkaline Phosphatase	339 IU/l
Total Bilirubin	3.7 mg/dl
Conjugated Bilirubin	1.4 mg/dl
Unconjugated Bilirubin	2.3 mg/dl
Serum Amylase	679 units/l
Serum Lipase	6149 units/l
INR (International normalized ratio)	1.29
Prothrombin time	18.6
APTT (Activated partial thromboplastin time)	38.4
Hepatitis B Surface Antigen	Negative
Hepatitis C Virus	Negative
HIV (Human Immunodeficiency Virus)	Negative

## Discussion

**Figure 3:**
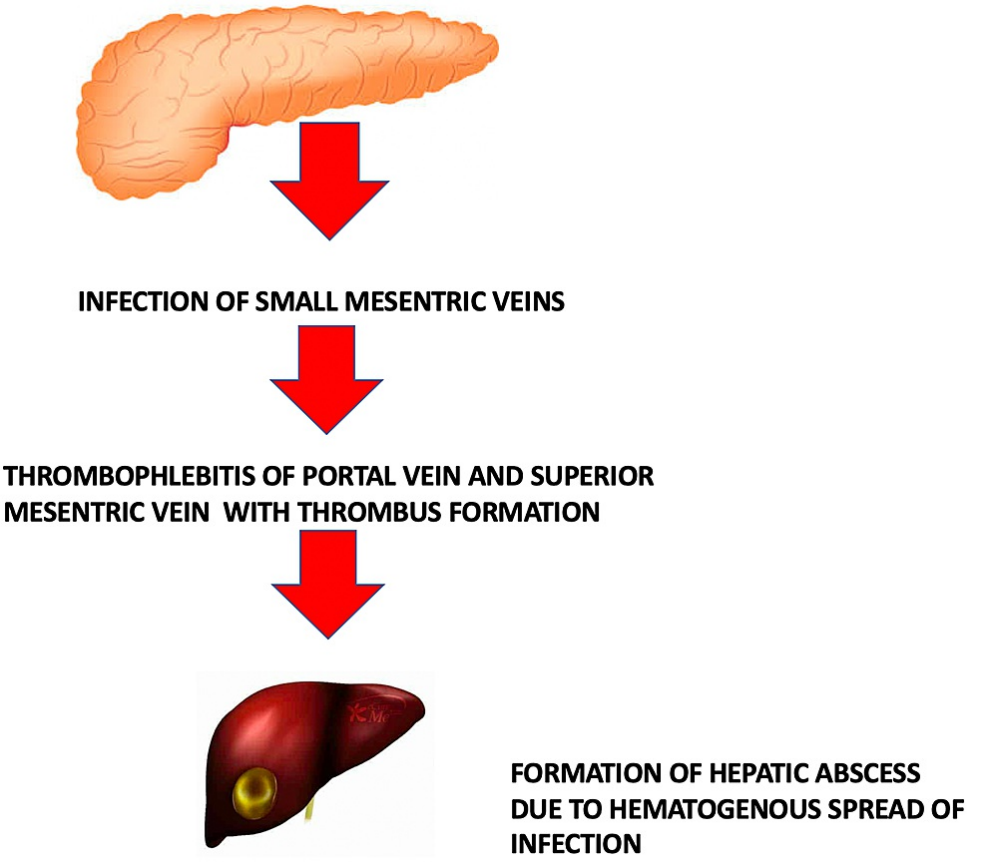
Pathophysiology of liver abscess formation in pylephlebitis

Pylephlebitis is the septic inflammation of the portal vein and or its hepatic branches. Initially, it starts as an infection of small veins dragging the territory of infection in the abdomen. The spread of this infection to larger veins ultimately leads to pylephlebitis, which can further lead to thrombosis of mesenteric veins (Figure 3). Presenting complaints most commonly remain fever and pain in the abdomen while malaise, diarrhea, anorexia, nausea and vomiting are also reported [6]. Jaundice can be a rare sign of an underlying hepatic abscess. This abscess can be visualized on imaging via computed tomography. Pylephlebitis manifests as enlargement and occlusion of the portal vein and its branches with intravascular thrombus formation. Cavernomatous transformation or periportal collaterals can develop in as few as 20 days following pylephlebitis. In our case, pylephlebitis further resulted in hepatic abscess formation, which can be seen as a cluster of small foci [7]. A pyogenic liver abscess occurs as a complication of hematogenous spread and therefore is multifocal as opposed to amoebic or other abscesses making the possibility of hematogenous spread of infection leading to hepatic abscess more likely in our case. During the early phase of abscess formation, the margins appear ill-defined with small non-enhancing cystic areas. Also, there is a heterogeneous enhancement of the liver parenchyma around the abscess. During the course of time, the abscess forms a well-circumscribed outline which has a thick enhancing rim in the periphery with fluid and debris lying internally [8]. The main differential diagnosis of portal vein thrombosis occurring with hepatic abscess is malignancy such as intrahepatic cholangiocarcinoma or hepatocellular carcinoma. Hepatocellular carcinoma is more common in a patient with other risk factors like hepatitis B or C or a patient with cirrhosis-hepatocellular carcinoma on imaging shows arterial enhancement with a delayed or portal washout. Intrahepatic cholangiocarcinoma shows arterial enhancement in the periphery, which is followed by an enhancement that is delayed progressive and centripetal. Hyperenhancement of intrahepatic cholangiocarcinoma, which is delayed, is thought to be due to the dense fibrous stroma of the tumor [9].

In a study carried out by Baril et al., it was found that patients with pylephlebitis had associated hypercoagulability [10]. A total of 18 patients out of 44 enrolled patients had associated hypercoagulable states which can be due to clotting factor deficiency or associated neoplasm. Our patient was also started on anticoagulant and is presently doing well on follow-up.

In our case, the absence of any imaging feature of malignancy with a clinical picture of fever with pain in the abdomen confirmed on lab investigations as sepsis with acute pancreatitis was suggestive of an intraabdominal source of sepsis with pylephlebitis leading to hepatic abscess as the diagnosis rather than malignancy. Pylephlebitis results as hematogenous dissemination of intraabdominal sepsis from locations drained by the portal system or splanchnic system. The most common etiology of pylephlebitis are diverticulitis, acute appendicitis, pancreatitis, inflammatory bowel disease and gastroenteritis. In our case, pylephlebitis and liver abscess were a complication of acute pancreatitis. The common cause of mortality is sepsis or peritonitis or bowel ischemia and infarction.

## Conclusions

Pyogenic liver abscess occurring as a complication of pylephlebitis due to intra-abdominal infection from acute pancreatitis is a rare but potentially fatal phenomenon. The treating physicians, along with respective radiologists, should therefore be alert and aware of such important but rare complications presenting with nonspecific complaints and no significant physical findings in order to prevent mortality and morbidity.
